# Analyses of odontogenic tumours: the most recent classification proposed by the World Health Organization (2017)

**DOI:** 10.4317/medoral.23751

**Published:** 2020-05-10

**Authors:** Kevin Barrios-Garay, Luisa-Fernanda Agudelo-Sánchez, José-Manuel Aguirre-Urizar, Cosme Gay-Escoda

**Affiliations:** 1 Dentistry student. Faculty of Medicine and Health Sciences, University of Barcelona, Barcelona, Spain; 2 MD, DDS, PhD. Chairman and Professor of Oral Medicine, Stomatology II Department, Faculty of Medicine and Nursery, University of the Basque Country/EHU, Leioa, Spain; 3 MD, DDS, MS, PhD, EBOS, OMFS. Chairman and Professor of Oral and Maxillofacial Surgery, Faculty of Medicine and Health Sciences, University of Barcelona. Director of the Master’s Degree Programme in Oral Surgery and Implantology (EHFRE International University/FUCSO). Coordinator/Researcher at the IDIBELL Institute. Head of the Oral Surgery, Implantology and Maxillofacial Surgery Department of the Teknon Medical Centre, Barcelona, Spain

## Abstract

**Background:**

The fourth edition of the ‘’WHO Classification of Head and Neck Tumours’’ was published in January 2017 and includes a classification of odontogenic tumours. This review aims to examine the changes made in this new classification in comparison with the previous classification of 2005.

**Material and Methods:**

An electronic search was conducted in the PubMed, Scopus and Cochrane databases with the keywords “odontogenic tumor”, “WHO classification” and “update”. Studies published from January 2009 to April 2019 with a high level of scientific evidence were included, but studies not published in English, epidemiological studies and studies with a low level of evidence were excluded.

**Results:**

The initial search found 457 articles and after eliminating duplicates, 8 studies were selected for full-text assessment. After excluding 3 epidemiological studies, 5 articles were finally included. These studies were stratified by their level of scientific evidence using SORT criteria (Strength of Recommendation Taxonomy).

**Conclusions:**

The new odontogenic tumour list has been simplified with the objective of improving its role as an international guide for diagnosis. Some changes have been possible thanks to the application of immunohistochemistry and molecular genetic techniques that allow better characterization of certain tumours. Further clinicopathological and molecular studies are needed so that this new classification can be consolidated and/or amended.

** Key words:**Odontogenic tumour, WHO classification, update.

## Introduction

Odontogenic tumours are defined as a neoplasms exclusively of the maxillary bones and oral mucosa, which derive from odontogenic epithelial tissue, odontogenic ectomesenchymal tissue or both (1). Odontogenic tumours represent less than 1% of all head and neck tumours. Benign tumours are more frequent and can occasionally show aggressive local growth and have a high recurrence rate (1).

In 1971, the World Health Organization (WHO) published the first odontogenic tumour classification with the aim of structuring and clarifying this type of pathology (2). This classification was revised in 1992 (3) and updated in 2005 (4). In January 2017, the ninth volume of the fourth edition of the WHO series was published, entitled ‘’WHO Classification of Head and Neck Tumours’’, following the consensus of various international experts. This monograph details odontogenic tumours alongside other tumours that affect the head and neck region.

The 2005 classification (4) needed a revision because some odontogenic tumour processes were explained unclearly, with confusing terminology and debaTable scientific evidence, which limited its use as a universal guide for diagnosis and management of odontogenic tumours (5). A significant change made in the 2005 classification (4) was the removal of the chapter on odontogenic cysts and the reclassification of some cystic lesions as keratocystic odontogenic tumours or calcifying cystic odontogenic tumours.

The main aim of the 2017 classification (1) has been to simplify the odontogenic tumour list proposed in 2005 (4) and to provide scientific evidence for each entity included in the new classification. In addition, in this edition, odontogenic cysts have been reincorporated, and these have been significantly updated since the 1992 classification (6,7).

The new odontogenic tumour classification distinguishes between benign and malignant tumours, as did the previous version (1). In 2005 (4), benign tumours were classified in three categories based on their origin: odontogenic epithelium with mature, fibrous stroma without odontogenic ectomesenchyme; odontogenic epithelium with odontogenic ectomesenchyme, with or without hard tissue formation; and mesenchyme and/or odontogenic ectomesenchyme with or without odontogenic epithelium. Despite the histological accuracy of these groups, the 2017 classification (1) has simplified them: epithelial odontogenic tumours, mixed epithelial and mesenchymal odontogenic tumours, and mesenchymal (ectomesenchymal) tumours.

Regarding malignant odontogenic tumours, both classifications maintain the distinction between carcinomas and sarcomas (1,4).

This review aims to analyse the main changes made in the new 2017 WHO odontogenic tumour classification (1) compared with the previous classification published in 2005 (4).

## Material and Methods

A bibliographical search was performed in the Cochrane, Scopus and PubMed-MEDLINE databases using the search terms “odontogenic tumour”, “WHO classification” and “update”. Search terms were combined using the Boolean operator “AND” with the aim of obtaining different articles that include two or more of the terms used for the search.

We included all studies published between January 2009 and April 2019 that were written in English and had level 1 or 2 scientific evidence according to SORT criteria (Strength of Recommendation Taxonomy) (8). Studies were excluded if they were not written in English, had an epidemiological aim or had level 3 scientific evidence according to SORT criteria (8).

## Results

**Figure 1:**
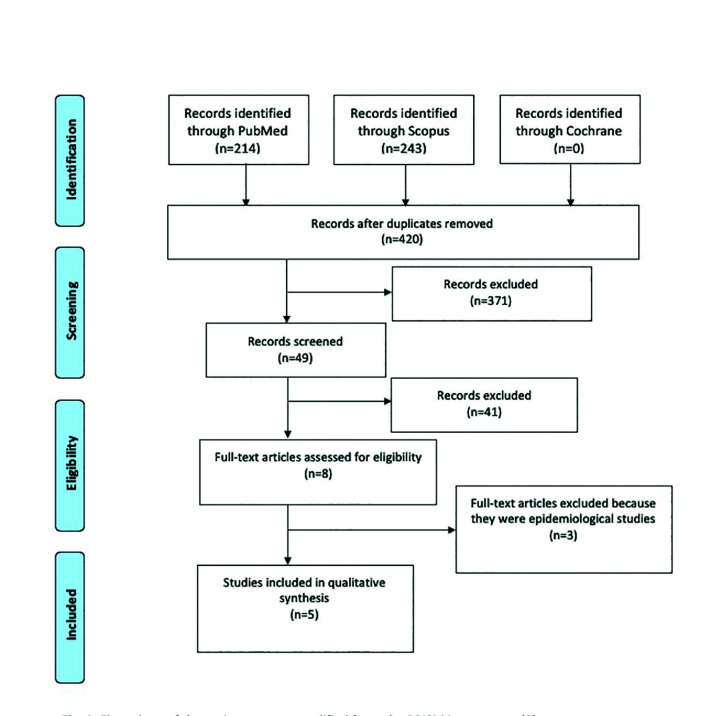
Flow-chart of the review process modified from the PRISMA statement.

We obtained 457 articles from the initial search in the different databases, and 420 after removing duplicates, corresponding to 214 studies from PubMed-MEDLINE, 243 from Scopus and none from Cochrane. 371 studies were then discarded after reading the title and the 49 remaining articles were screened by reading the abstract. Of these, 41 were discarded because they only concerned odontogenic cysts or odontogenic tumour treatment or did not contribute any relevant information relating to the new classification. From the remaining 8 articles assessed for full-text eligibility, 3 studies were excluded because they were epidemiological studies and did not meet the inclusion criteria. Finally, 5 studies were included for this review: three systematic reviews, one retrospective study and a review. Fig. 1 shows the flow-chart of the review process under PRISMA criteria (9).

The studies were stratified according to their scientific evidence level using SORT criteria (8), resulting in 3 articles with level 1 evidence and 2 with level 2 evidence.

First of all, it is recognized that the malignant tumour list presented in the new classification has been simplified compared with the previous one. The list of tumours is reduced from 12 entities in 2005 to 7 entities in 2017 (Table 1). As a consequence of this simplification, there is only one type of ameloblastic carcinoma and one type of primary intraosseous carcinoma, as tumour entities with adjectives such as secondary, not differentiated, etc. have been deleted. In addition, this new edition proposes the acknowledgment of only one type of odontogenic sarcoma (1).

The new classification also acknowledges one new type of malignant tumour, sclerosing odontogenic carcinoma, based on previous publications concerning this tumour (10-12).

Regarding benign tumours, the 2005 classification has likewise been simplified with the disappearance of some entities (Table 2). There is also the addition of a new benign neoplasm, the primordial odontogenic tumour, described and categorized in 2014 (13).

With odontogenic cysts incorporated into the new 2017 WHO text (1), keratocystic odontogenic tumour and calcifying epithelial odontogenic tumour are once again considered as cystic entities, respectively odontogenic keratocyst and calcifying epithelial cyst.

**Table 1:**
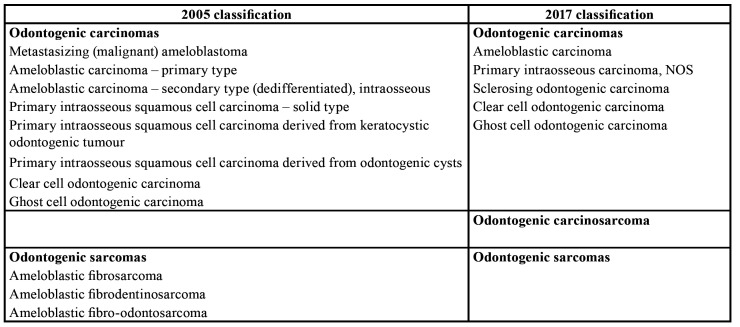
Comparison of malignant odontogenic tumours.

**Table 2:**
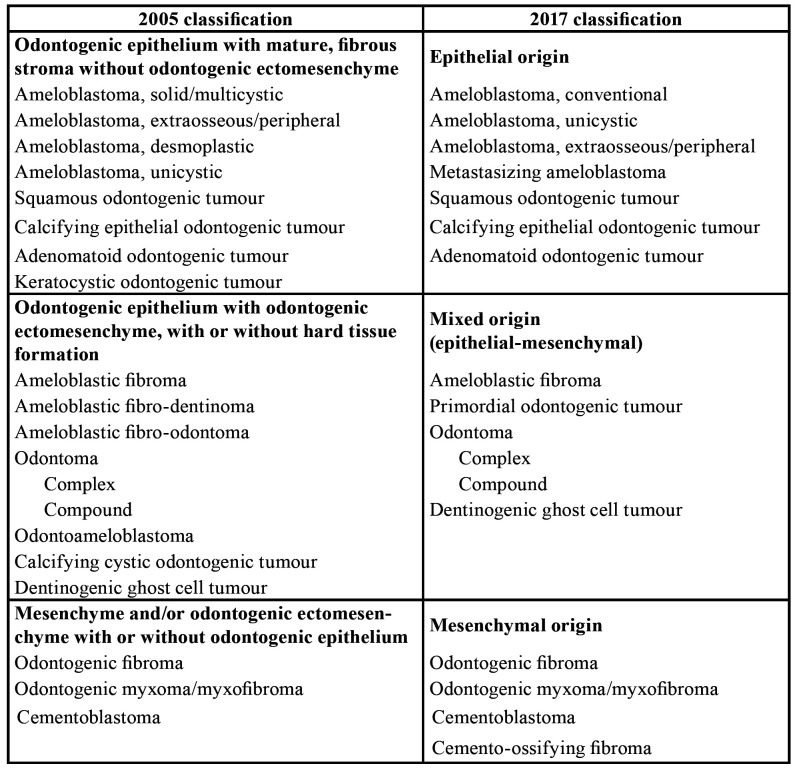
Comparison of benign odontogenic tumours.

## Discussion

- Malignant odontogenic tumours

Ameloblastic carcinoma was divided in the 2005 classification into three categories: primary, secondary intraosseous and secondary peripheral. In this new classification, it was decided to delete these categories because they had very similar morphology and clinical behaviour (6). This malignant tumour shares the BRAF V600E mutation with its benign counterpart, ameloblastoma. When diagnosing this tumour, sex-determining region Y-related high mobility group box 2 (SOX2) and high proliferation index (Ki-67) are necessary to distinguish it from ameloblastoma (14,15).

Primary intraosseous carcinoma is a single entity in this edition (1), unlike in the previous classification (4) where it was divided into three categories based on its histogenesis.

Sclerosing odontogenic carcinoma is a new malignant tumour included in this classification (1) after being described by Koutlas *et al* in 2008 (10) and with more than 10 cases reported (10-12). It is a primary intraosseous carcinoma with an epithelial component with low-level atypia and pleomorphism within sclerotic connective stroma that can infiltrate muscle and nerve structures (1). It has not yet been clarified whether it constitutes a specific entity or a particular histomorphological pattern, and more extensive tumoral characterization is necessary to justify this tumour as a unique entity (6,15).

There have been no major changes to clear cell odontogenic carcinoma in this new classification, beyond immunohistochemistry and genetic characterization (1). Proliferation index (Ki-67) greater than 20% and positive MMP-9 (metalloproteinase 9) differentiate it from other entities that are morphologically similar (15,16). 80% of these carcinomas also show rearrangements in the EWSR1-ATF genes, although these rearrangements also appear in salivary gland hyalinizing clear cell carcinoma and other clear cell neoplasms (17).

A new malignant tumour entity, odontogenic carcinoma with dentinoid, was described for the first time in 2014 (18). Nevertheless, it was decided not to include this in the 2017 classification (1) because it was felt that there was not enough scientific evidence and that more time was needed to allow for additional new cases to be published. This carcinoma is being discussed in relation to clear cell odontogenic carcinoma, which can show dentinoid in 7% of cases (6).

Odontogenic carcinosarcoma is an entity that was first described in the 1992 classification (3) and was deleted in the 2005 edition (4) because it had insufficient characterization. After new cases were published, it was decided to reinclude this tumour in the 2017 classification (19).

Regarding odontogenic sarcomas, in the previous edition (4) they were classified as ameloblastic fibrosarcoma, ameloblastic fibrodentinosarcoma or ameloblastic fibro-odontosarcoma. This classification was based on the presence of different hard dental tissue types and quantities (4). It has been stated that the most common type is ameloblastic fibrosarcoma (6) but the authors of the new classification consider that there is not enough scientific evidence to subclassify these odontogenic sarcomas (1). This simplification is not universally agreed and controversies may develop when new cases are published.

- Benign odontogenic tumours

a) Epithelial origin

There have been changes to the terminology and classification of ameloblastomas based on new discoveries about the genetic profile of this tumour (1). In the previous edition (4), ameloblastomas could be classified as solid / multicystic, peripheral / extraosseous, desmoplastic or unicystic. In this new edition, it was decided to delete the ‘‘solid / multicystic’’ adjective because it had little biological value and could lead to confusion with ‘’unicystic’’ ameloblastoma (6). However, Kennedy (7) points out that pathologists continue to use these adjectives to differentiate conventional ameloblastoma (solid) from unicystic and other variants. In this edition, desmoplastic ameloblastoma is reclassified as a histopathological variant because despite its particular radiographic and clinical features, it behaves like any other ameloblastoma (6). Between the two classifications, various genetic mutations have been discovered in ameloblastomas. In 90% of these tumours, mutations in the MAPK pathway are found, with BRAF V600E being the most common mutation, whilst outside the MAPK pathway SMO mutation is the most frequently referenced (20-22).

Regarding unicystic ameloblastoma, there are currently three histopathological types: luminal, intraluminal and mural. The mural variant is controversial since it is considered to be more of a conventional ameloblastoma with cystic degeneration rather than a true unicystic ameloblastoma (6). As in conventional ameloblastoma, BRAF V600E mutations have been detected (23).

Metastasizing ameloblastoma is an odontogenic tumour that was classified in 2005 (4) as a malignant tumour. In this new edition (1), it has been decided to classify it as a benign tumour due to the benign histologic appearance of both primary tumour and metastases. The most frequent metastases are in the lungs, followed by lymph nodes and bone (1,24).

In aggressive relapsing and/or metastatic ameloblastoma cases, apart from surgery, anti-BRAF biotherapy can be used. These are tyrosine kinase inhibitors (vemurafenib or dabrafenib + trametinib) (25,26).

There have been no major changes to squamous odontogenic tumour, calcifying epithelial odontogenic tumour or adenomatoid odontogenic tumour (1).

In 2005 (4), odontogenic cysts were not included in the classification and odontogenic keratocyst was listed as “keratocystic odontogenic tumour” inside the epithelial-origin benign tumours category. In 2017 (1), with the reincorporation of odontogenic cysts, it has been reclassified again as odontogenic keratocyst.

b) Epithelial-mesenchymal origin

Odontoameloblastoma, which has ameloblastic proliferation and dental tissue formation, was added to this group in the 2005 classification (4). In the 2017 classification (1), it was considered that this neoplasm does not constitute a unique entity because there is not enough evidence that these tumours originate as odontoameloblastomas and that after being removed they arise again as odontoameloblastomas (6,7).

In the new edition (1), calcifying odontogenic tumour is once again listed as calcifying odontogenic cyst, because there is not enough scientific evidence to justify considering this entity a neoplasm (6).

In 2005 (4), ameloblastic fibroma could have two variants depending on which hard dental tissue it presented: ameloblastic fibro-dentinoma and ameloblastic fibro-odontoma. In this new edition (1), it has been decided to delete these variants because it was felt that once these tumours develop they will turn into odontomas, so in the experts’ opinion there was little evidence to justify keeping them as separate entities (6,27).

The first 6 cases of primordial odontogenic tumour were published in 2014 (13). This tumour is histologically composed of variably cellular loose fibrous tissue with areas similar to the dental papilla, entirely surrounded by cuboidal to columnar epithelium resembling the internal epithelium of enamel origin.

There have been no major changes to the odontoma group in this new edition (1) compared with the previous one (4).

Ghost cell dentinogenic tumour has been updated as well as its immunohistochemistry profile (1).

c) Mesenchymal origin

The previous edition (4) listed two histopathological types of odontogenic fibroma: simple and complex. This depended on whether the tumour was epithelium poor (simple) or epithelium rich (complex). In this edition, it was decided to delete this differentiation because it was poorly documented (6).

Neither odontogenic myxoma/myxofibroma nor cementoblastoma have undergone any substantial changes in this new edition (1).

Cemento-ossifying fibroma is a neoplasm that was not included in the 2005 edition (4) but has been added to the 2017 classification (1) under mesenchymal-origin odontogenic tumours, in order to distinguish cemento-ossifying from juvenile forms (trabecular and psammomatoid), remarking on its odontogenic origin because it is believed to arise from periodontal tissue. This entity is detailed in the monograph with ossifying fibromas in the fibro-osseous and osteochondromatous section (1).

## Conclusions

The current WHO odontogenic tumours classification has been simplified in comparison with the 2005 edition with the aim of improving its use as an international guide for the diagnosis and management of this pathology.

Many of these changes in classification have been possible thanks to better genetic and immunohistochemistry characterization of some tumours, which was not possible in the last edition.

It is important to point out that this classification process is far from complete because there are entities such as metastasizing ameloblastoma and sclerosing odontogenic carcinoma that do not yet have enough scientific evidence to be characterized appropriately. In this regard, we would find odontogenic carcinoma with dentinoid as well as another evaluation of tumoral entities deleted in this classification.
